# Dr. B. A. Satterthwaite,—Lima, O.

**Published:** 1853-01

**Authors:** 


### DR. B. A SATTERTHWAITE,—LIMA, O.

This gentleman, one of the first graduates from the Ohio College of Dental Surgery, we recollect, when preparing a specimen set of teeth for the Museum of the Institution, took all the materials in their rough state, making his teeth (all common plate teeth without gum) and everything else requisite to complete the operation. We are reminded of this fact, by receiving from him a few days since, a present of two sets of teeth—one gum and the other plain. These teeth taken throughout, are as near the shape of the natural organs as any we have ever seen, and evidently have passed more carefully under the carving knife than is the ordinary custom. The cusps, the depressions, and all the marked characteristics are truly delineated. The coloring of the gum and teeth are also very good, and in the mouth would present a very natural appearance. We are informed by the doctor, that he does not expect to manufacture them to any extent for sale. We are pleased to see the perfection to which the doctor has brought this part of the dental art. We believe there are few who pretend to make their own teeth. But few have the patience and application to succeed.

